# A New Method to Perform Direct Efficiency Measurement and Power Flow Analysis in Vibration Energy Harvesters

**DOI:** 10.3390/s21072388

**Published:** 2021-03-30

**Authors:** Jan Kunz, Jiri Fialka, Stanislav Pikula, Petr Benes, Jakub Krejci, Stanislav Klusacek, Zdenek Havranek

**Affiliations:** 1 Department of Control and Instrumentation, Faculty of Electrical Engineering and Communications, Brno University of Technology, Technicka 3082/12, 616 00 Brno, Czech Republic; xkunzj00@vutbr.cz (J.K.); jiri.fialka@ceitec.vutbr.cz (J.F.); pikula@vutbr.cz (S.P.); xkrejc44@vutbr.cz (J.K.); klusacek@vutbr.cz (S.K.); zdenek.havranek@ceitec.vutbr.cz (Z.H.); 2 CEITEC—Central European Institute of Technology, Brno University of Technology, Purkynova 656/123, 612 00 Brno, Czech Republic

**Keywords:** piezoelectric, piezoelectric ceramic, lead zirconate titanate (PZT), polyvinylidene fluoride (PVDF), energy harvesting, efficiency, efficiency measurement, power conversion, power flow

## Abstract

Measuring the efficiency of piezo energy harvesters (PEHs) according to the definition constitutes a challenging task. The power consumption is often established in a simplified manner, by ignoring the mechanical losses and focusing exclusively on the mechanical power of the PEH. Generally, the input power is calculated from the PEH’s parameters. To improve the procedure, we have designed a method exploiting a measurement system that can directly establish the definition-based efficiency for different vibration amplitudes, frequencies, and resistance loads. Importantly, the parameters of the PEH need not be known. The input power is determined from the vibration source; therefore, the method is suitable for comparing different types of PEHs. The novel system exhibits a combined absolute uncertainty of less than 0.5% and allows quantifying the losses. The approach was tested with two commercially available PEHs, namely, a lead zirconate titanate (PZT) MIDE PPA-1011 and a polyvinylidene fluoride (PVDF) TE LDTM-028K. To facilitate comparison with the proposed efficiency, we calculated and measured the quantity also by using one of the standard options (simplified efficiency). The standard concept yields higher values, especially in PVDFs. The difference arises from the device’s low stiffness, which produces high displacement that is proportional to the losses. Simultaneously, the insufficient stiffness markedly reduces the PEH’s mechanical power. This effect cannot be detected via the standard techniques. We identified the main sources of loss in the damping of the movement by the surrounding air and thermal losses. The latter source is caused by internal and interlayer friction.

## 1. Introduction

The increasing demands on the monitoring and predictive maintenance of mechanical structures, intelligent building control, and other remote sensing applications generate the need to install electronics at isolated locations. Such sites are often characterized by difficult access and a lack of infrastructure. Self-powered electronic devices thus became an essential prerequisite. These instruments and apparatuses gather electricity from sources such as light, temperature difference, and diverse forms of kinetic energy, including ambient mechanical vibrations [1,2]. The vibrations can be converted to usable electrical energy by means of piezoelectric energy harvesters (PEHs).

This type of movement is present in machines and structures where collecting relevant operational information has a beneficial impact, namely in industrial equipment, cars, aircraft, and bridges [1,3]. Moreover, PEHs are also employed in harvesting from human movement [1]; substantial effort has therefore been invested in developing and improving PEHs to generate as much energy as possible from the oscillations.

The power output constitutes the key criterion to determine the applicability of a PEH; however, this factor does not provide sufficient information if individual PEHs are to be compared. The reason is that the maximum power output is defined mainly by the PEH’s dimensions and vibration amplitude. In view of these facts, Beeby et al. [4] proposed normalized power density (NPD), which divides the power output by the vibration amplitude and the volume of the harvester. Although such metrics appear suitable for comparing PEHs, the authors also mention certain drawbacks. One of the disadvantages rests in ignoring important factors, including the bandwidth [4]. Generally, however, Beeby et al.’s approach is simple, straightforward, and easily applicable. Other researchers developed more sophisticated methodologies, involving the frequency parameters; these techniques were analyzed comprehensively by Hadas et al. [5], who outlined their benefits and drawbacks.

A portion of relevant papers interpret the PEH as an energy converter, meaning that the power output and related alternatives are not considered convenient for a comparison of PEHs. The authors thus employ efficiency as the criterion to examine the devices, as is common in other energy converters. The relevant theories and measurement procedures were summarized and discussed by Yang et al. [6]. In their paper [6] the efficiency not only embodies an essential factor in the development and optimization of PEHs but also finds use as a parameter allowing comparison between energy harvesting methods.

### Efficiency

The efficiency is the ratio between the power output and input. In PEHs, the electrical power output is measurable without difficulty. Determining the mechanical power input, however, constitutes a more challenging task, as the process cannot be easily monitored [6]. To establish the efficiency, the power input of the harvester is often simplified to its mechanical power [6,7] because this quantity can be easily calculated via the PEH’s parameters.

The calculation is performed by means of, for example, the widely applied formula proposed by Richards et al. [7], which defines the efficiency for the resonant frequency and optimal load [6]:

(1) $$\eta =\frac{1}{2}\frac{k_{\mathrm{sys}}^{2}}{1-k_{\mathrm{sys}}^{2}}/\left(\frac{1}{Q}+\frac{1}{2}\frac{k_{\mathrm{sys}}^{2}}{1-k_{\mathrm{sys}}^{2}}\right),$$

where η is the efficiency, and $k_{\mathrm{sys}}$ and *Q* denote the system coupling coefficient and the quality factor of the PEH, respectively.

Some of the researchers [6,8,9] expanded the efficiency calculation to involve broader frequency and load ranges. Regrettably, neither the calculating process nor the measurement of the parameters are unified, resulting in that the determined efficiencies oscillate between less than 1% [10,11] and more than 80% [8,12] in lead zirconate titanate (PZT) PEHs.

The role of the system coupling coefficient ($k_{\mathrm{sys}}$) is not identical with that of the electromechanical coupling coefficient ($k_{\mathrm{eff}}$) defined in [13,14]. The latter embodies a piezomaterial coefficient, whereas the former ($k_{\mathrm{sys}}$) describes the whole structure of the PEH. The material coupling coefficient can be obtained from the material resonance and anti-resonance frequencies [13]:

(2) $$k_{\mathrm{sys}}^{2}=\frac{f_{o}^{2}-f_{s}^{2}}{f_{o}^{2}},$$

where $f_{o}$ and $f_{s}$ are the open and the short natural frequencies of the PEH, respectively.

Equation (2) has found wide use [6,7,8], in spite of the fact that it is valid only for undamped systems (damping coefficient, ζ=0) [15,16]. Utilizing Formula (2) in damped systems (ζ>0) produces inaccurate parameters [15] and, consequently, imprecise efficiency.

Regrettably, this method (Equation (2)) for calculating the $k_{\mathrm{sys}}$ exhibits significant uncertainty due to two large numbers being subtracted close to each other; therefore, the efficiency calculation (Equation (1)) will comprise a major error, too. This error, however, is not evaluated in the literature [6,7,10,11,17,18].

Some experts [6,12] also measured experimentally the mechanical power of PEHs. Although such an attitude finds application in validating the derived formulas, it still ignores the mechanical losses. By extension, the parameters of the devices have to be employed to calculate the power. This simplification assumes that the ambient vibration source has an “infinite power” compared to the consumed power of the PEH. The mechanical losses can therefore be ignored, as they are easily replenished from the source [19]. In some situations, such as harvesting from human motion, this assumption is invalid, and the mechanical losses have to be taken into account [19]. Furthermore, the mechanical losses also constitute an important parameter in developing the PEH.

Liao and Sodano [20] established that the approach neglecting the mechanical losses is oversimplified, and they proposed that the input power should not be considered the mechanical power of the PEH. The authors recommend using the power fed into the device from the ambient source to maintain the steady state vibrations. Although this claim is correct, the calculation of the power from the parameters of the PEH cannot be characterized in the same manner. The reason rests in that the procedure involves only the internal and electrical losses and ignores the mechanical ones (e.g., the internal friction or aerodynamic drag). In this context, Yang et al. [6], for example, found close similarity between their method and the PEH’s damping coefficient.

Based on our review of the literature, we can conclude that the mechanical losses are disregarded in the current efficiency measurements, which exploit the mechanical power of PEHs as the input. Furthermore, although the harvesters’ parameters are utilized for the calculations, the measurement method and the uncertainties are either characterized unclearly or remain ignored [6,8,10,11].

Such issues then justify the need for a new measurement system capable of evaluating the efficiency via the mechanical power supplied to a PEH by an ambient vibration source (proposed efficiency). This power is determined from the force and velocity of the source (in our case, a vibration shaker), eliminating the necessity to use the parameters of the PEH in the calculation. Furthermore, the same measurement assesses the harvester’s mechanical power, too, and the efficiency can then be established in a simplified manner (simplified measured). Importantly, the device’s parameters ($k_{\mathrm{sys}}$ and *Q*) are calculable from the results to facilitate computing the efficiency according to (1) (simplified calculated). In addition, the uncertainties of all of the aforementioned approaches are determined during the measuring procedure.

As indicated, the unique setup allows us to yield the proposed efficiency, the simplified efficiencies, and the related uncertainties from merely a single measurement. The methods are thus compared in identical conditions. Expectably, the simplified efficiencies, being comparable, should exhibit the same values. The proposed efficiency nevertheless shows lower values, as it includes the mechanical losses.

The paper is organized as follows: The introductory sections set out the problem, together with relevant processes, procedures, and options. Section 2 debates the methodology, characterizing the power flow in piezoelectric harvesters, outlining the calculation of the input power, and describing the measurement system and its uncertainties. In Section 3, the main discussion and results are presented, including the outcomes obtained from a measurement of the efficiency of two different commercially available harvesters. The results of the proposed method are correlated to common simplified efficiencies. Finally, the power flow is discussed, in conjunction with a quantitative analysis of the losses.

## 2. Methods

**Figure 1:**
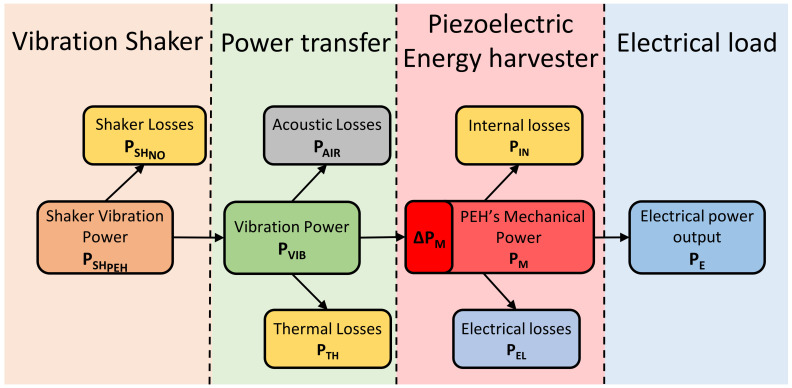
The power flow in a piezo energy harvester (PEH). The traditional approach to determine the efficiency considers the harvester’s mechanical power ($P_{M}$) as the input power. Alternatively, according to the definition, the power fed into the PEH from the ambient source ($P_{\mathrm{VIB}}$) has to be used as the input.

Prior to describing the efficiency measurement system, we will discuss the power flow in PEHs (Figure 1). During each cycle, the energy is transmitted back and forth due to the oscillations of the system. In this chapter, however, only the power flow average through several cycles is assumed.

In a laboratory environment, the vibrations are generated by a vibration exciter (shaker), which produces a vibration power ($P_{{\mathrm{SH}}_{\mathrm{PEH}}}$) and has its own losses ($P_{{\mathrm{SH}}_{\mathrm{NO}}}$).

The power from the vibration source ($P_{\mathrm{VIB}}$) is transferred to the vibration movement of the PEH, i.e., the harvester’s mechanical power ($P_{M}$). Similarly to every other power transfer, this process generates some losses. In the presented case, the two main sources are the thermal losses ($P_{\mathrm{TH}}$) generated by the internal friction in the PEH, and the acoustic losses ($P_{\mathrm{AIR}}$), originating from the movement of the PEH in the air.

The mechanical power ($P_{M}$) is further transferred to the harvester’s usable electrical power output ($P_{E}$). Here, the main sources of loss are the internal losses ($P_{\mathrm{IL}}$), arising mainly from the electromechanical losses in the piezoelectric and electrical dissipation ($P_{\mathrm{EL}}$) on the PEH’s internal resistance.

In steady state vibrations, however, only a part of the PEH’s mechanical power ($\Delta P_{M}$) is dissipated in the form of the internal and electrical losses. The electrical power output is replenished from the vibration source, as proposed by [20]. Nevertheless, the amount of such replenished power is difficult to evaluate. For simplification we assume in the calculation that the entire mechanical power of the PEH is replenished from the vibration source. This approach also allows us to be consistent with other efficiency-related papers, except for [20]. Similarly to our procedure, however, the authors of this referenced source, too, were unable to measure the losses.

The applied simplification underestimates the thermal losses ($P_{\mathrm{TH}}$) as these are calculated via the PEH’s mechanical power ($P_{M}$). The efficiencies are not affected, because the traditional approach to determine the efficiency employs the mechanical power ($P_{M}$) as the input power [6]. However, according to the definition [20], the power supplied to the PEH from the ambient source, namely the $P_{\mathrm{VIB}}$ in our case, has to be used as the input. For clarification purposes, the methods to measure or calculate the powers expressed in Figure 1 are described in the sections below.

### 2.1. Shaker Vibration Power

The vibration power of the shaker ($P_{\mathrm{SH}}$) is calculated out of the known force and velocity (Equation (3)) [6,18,21]. As both of the quantities are vectors, their phases have to be measured, too. In the steady state condition and at harmonic vibration, the power of the mechanical vibration is calculated as:

(3) $$p_{\mathrm{SH}}=F\cdot v=|F||v|\cos\left(\phi \right).$$

The velocity of the structure’s base is either directly measured or, as in our case, calculated from the known acceleration. Since the vibrations are steady state and harmonic, the calculation simplifies into:

(4) $$\left|v\right|=\left|\int adt\right|=\int a_{\mathrm{ampl}}\sin\left(2\pi ft+\phi_{a}\right)dt=\frac{a_{\mathrm{ampl}}}{2\pi f}\cos\left(2\pi ft+\phi_{a}\right).$$

The electrodynamic shaker is, principally, a loudspeaker. To calculate the force generated by the shaker, we can then employ the formula of Ampere’s law, commonly used for this purpose in acoustics [22]:

(5) F=lI×B.

As the magnetic flux density *B* in the coil gap and the length of the wire *l* in the magnetic field remain constant, their product, $k_{Bl}$, can be easily determined [22], simplifying Equation (5) to read

(6) $$\left|F\right|=k_{Bl}\left|I\right|=k_{Bl}I_{\mathrm{ampl}}\sin\left(2\pi ft+\phi_{I}\right).$$

Substituting (4) and (6) into (3) and averaging the outcome through the period, we yield:

(7) $$\begin{array}{c} P_{\mathrm{SH}}=\frac{1}{T}\int_{T}p_{\mathrm{SH}}dt=\frac{1}{T}\int_{T}k_{Bl}I_{\mathrm{ampl}}\sin\left(2\pi ft+\phi_{I}\right)\frac{a_{\mathrm{ampl}}}{2\pi f}\cos\left(2\pi ft+\phi_{a}\right)dt\\ =k_{Bl}I_{\mathrm{rms}}\frac{a_{\mathrm{rms}}}{2\pi f}\cos\left(\phi -\frac{\pi }{2}\right), \end{array}$$

where $p_{\mathrm{SH}}$ and $P_{\mathrm{SH}}$ are the shaker’s instantaneous and average vibration power, respectively; $F_{\mathrm{rms}}$ and $v_{\mathrm{rms}}$ are the effective values of the vibration force and velocity, respectively; $k_{Bl}$ denotes the shaker’s Bl coefficient; $I_{\mathrm{rms}}$ and $a_{\mathrm{rms}}$ are the effective values of the drive current and acceleration, respectively; ϕ is the phase between those two vectors; and *f* represents the frequency of the vibrations.

### 2.2. Vibration Power

The measurement of the vibration power ($P_{\mathrm{VIB}}$) delivered from the vibration source to the PEH is rather complicated [6]. In the case of steady state harmonic vibrations, however, the power can be determined from the vibration power of the shaker ($P_{\mathrm{SH}}$).

The power generated by the vibration shaker ($P_{\mathrm{SH}}$) depends on the vibration amplitude, frequency, and load on the vibration rod and fixture. Advantageously, the power to vibrate structures other than the PEH is measurable as well, by the same measurement but without the PEH mounted. Then, the vibration power ($P_{\mathrm{VIB}}$) can be calculated simply by subtracting the vibration power without the PEH ($P_{{\mathrm{SH}}_{\mathrm{NO}}}$) from that with the device ($P_{{\mathrm{SH}}_{\mathrm{PEH}}}$). We have:

(8) $$P_{\mathrm{VIB}}=P_{{\mathrm{SH}}_{\mathrm{PEH}}}-P_{{\mathrm{SH}}_{\mathrm{NO}}},$$

where $P_{\mathrm{VIB}}$ is the vibration power consumed by the PEH from the ambient vibration source, and $P_{{\mathrm{SH}}_{\mathrm{PEH}}}$ and $P_{{\mathrm{SH}}_{\mathrm{NO}}}$ stand for the shaker’s vibration power with and without the PEH mounted, respectively.

### 2.3. Acoustic Losses

The motion of the piezoelectric cantilever is damped by the ambient air. The damping produces an acoustic power, calculable from the known deflection of the moving object. The acoustic power for a harmonic oscillation is given by [22], as follows:

(9) $$P_{\mathrm{air}}=AI=A\frac{1}{2}\rho c\omega^{2}d^{2},$$

where *A* is the area, *I* denotes the sound intensity, ρ represents the air density, *c* is the speed of sound in the air, ω stands for the angular frequency, and *d* is the oscillation amplitude.

Due to the nature of the cantilever deflection, the calculation can be materialized when the resonance mode is measured at *n* points evenly distributed on the surface of the cantilever. The measured deflections ($d_{i}$) are applicable in establishing the partial intensities ($I_{i}$), and we sum them to estimate the total acoustic power; we then have:

(10) $$P_{air_{tot}}=\sum_{i=1}^{n}\frac{A}{n}I_{n}=\sum_{i=1}^{n}\frac{A}{n}\frac{1}{2}\rho c\omega^{2}d_{i}^{2}.$$

When the deflections are measured on the edge of the structure, this arrangement has to be taken into account and then reflected in the calculation by an appropriate reduction of the corresponding area.

### 2.4. Thermal Losses

The power transfer from the vibration rig to the movement of the PEH, the PEH’s mechanical power ($P_{M}$), involves losses, such as the internal friction between and in the material layers of the harvester. The losses dissipate some of the power to heat. By extension, in more general terms, losses also accompany the mechanical–electrical power conversion in the piezoelectric (Section 2.8) and the electrical losses (Section 2.7) on the internal resistance; both are also dissipated as heat. Thus, the powers of the individual loss components cannot be evaluated by measuring the heat.

Moreover, in PEHs, the overall power dissipated into heat is so small that it produces only an almost insignificant change in the harvester’s surface temperature, for instance, in the PZT PEH at 1 $g_{\mathrm{rms}}$ the warming of the PEH caused by the thermal losses (14.93 mW; Table 1) is less than 0.02 K. For this reason, we were unable to measure the outcome.

Beneficially, the amount of thermal losses ($P_{\mathrm{TH}})$ can be determined in an indirect manner, via:

(11) $$P_{\mathrm{TH}}=P_{\mathrm{VIB}}-P_{\mathrm{AIR}}-P_{M},$$

where $P_{\mathrm{TH}}$ and $P_{\mathrm{AIR}}$ are the thermal and the acoustic losses, respectively, and $P_{\mathrm{VIB}}$ and $P_{M}$ denote the vibration and the mechanical powers of the PEH, respectively.

### 2.5. PEH Mechanical Power

The mechanical power of a PEH ($P_{M}$) can be measured via Formula (12), derived by Yang et al. [6]:

(12) $$P_{M}=\frac{1}{2}m_{e}a_{X}v_{Z}\sin\left(\phi_{X}\right),$$

where $m_{e}$ is the PEH’s effective mass, $a_{x}$ denotes the amplitude of the relative acceleration of the PEH’s tip, $v_{Z}$ represents the amplitude of the base velocity, and $\phi_{X}$ is the phase difference between the base velocity and the PEH’s tip acceleration.

### 2.6. Electrical Power Output

The electrical power output of the PEH ($P_{E}$) is measured on the resistive load; however, it alternates with the vibration frequency. Thus, the output has to be rectified to be able to power some types of electronics, and additional losses occur. For this reason, rectification was not involved in our experiments. Relevant power management circuits are also being intensively developed [5]; these are nevertheless not discussed in this paper. We have:

(13) $$P_{E}=\frac{U_{\mathrm{rms}}^{2}}{R_{L}},$$

where $P_{E}$ is the PEH’s electrical power output, and $U_{\mathrm{rms}}$ denotes the effective value of the voltage generated by the PEH on the preset resistive load, $R_{L}$.

### 2.7. Electrical Losses

To ensure the maximum flow of power from the PEH to the load, the optimum external load has to have the same value as the internal resistance of the PEH. Then, the power transfer is at a maximum but provides an efficiency of only 50%, meaning that half of the generated electrical power is the electrical power output ($P_{E}$) and the other half comprises the electrical losses ($P_{\mathrm{EL}}$) dissipated on the internal resistance. Then, the electrical losses ($P_{\mathrm{EL}}$) can be easily determined:

(14) $$P_{\mathrm{EL}}=P_{E},$$

where $P_{\mathrm{EL}}$ denotes the electrical losses on the internal resistance, and $P_{E}$ is the electrical power output.

### 2.8. Internal Losses

The mechanical–electrical power conversion is performed by the piezoelectric layer in the PEH. The portion of the mechanical power that is absorbed by the layer but remains unconverted into the electrical power ($P_{E}$) forms the internal losses ($P_{\mathrm{IL}}$), which are dissipated into heat.

The efficiency of the conversion in the piezomaterial is defined by the electromechanical coupling coefficient ($k_{\mathrm{eff}}$). The relevant value usually varies between 0.4 and 0.6 in the PZT ceramic but amounts to less than 0.3 in the polyvinylidene fluoride (PVDF) film. The conversion efficiency of the material enclosed in the PEH’s structure is slightly different, and therefore the internal losses ($P_{\mathrm{IL}}$) cannot be calculated directly by using this coefficient. However, indirect calculation is possible; we then have:

(15) $$P_{\mathrm{IL}}=P_{M}-P_{E}-P_{\mathrm{EL}},$$

where $P_{\mathrm{IL}}$ and $P_{\mathrm{EL}}$ are the internal and electrical losses, respectively, and $P_{M}$ and $P_{E}$ represent the mechanical and electrical powers, respectively.

### 2.9. Efficiency Calculation

To include the mechanical losses in the efficiency, we consider as an input power the vibration power supplied to the PEH from an ambient vibration source, $P_{\mathrm{VIB}}$. We have:

(16) $$\eta =\frac{P_{E}}{P_{\mathrm{VIB}}}=\frac{\frac{U_{\mathrm{rms}}^{2}}{R_{L}}}{P_{{\mathrm{SH}}_{\mathrm{PEH}}}-P_{{\mathrm{SH}}_{\mathrm{NO}}}},$$

where η stands for the measured efficiency; $P_{\mathrm{VIB}}$ is the PEH’s power input; $P_{E}$ denotes the electrical power output, i.e., the voltage *U* generated across the load resistor, $R_{L}$; and $P_{{\mathrm{SH}}_{\mathrm{PEH}}}$ and $P_{{\mathrm{SH}}_{\mathrm{NO}}}$ represent the vibration power with and without the PEH mounted on the shaker, respectively.

### 2.10. Efficiency Measurement

To measure the efficiency, we improved the automated measurement system [23] to manage the vibration amplitudes at a lower error rate and to reduce the noise. Moreover, the data post-processing stage was redesigned to compute the power consumption of the PEH. The actual setup is described in detail within Section 2.12.

Because the efficiency results appear to be the most interesting near the PEH’s mechanical resonance, this resonance is localized by using sweep sine vibration.

To obtain proper efficiency results, we need to ensure that the PEH vibrates in its first longitudinal mode without any torsional oscillation or clamping. Such a condition was verified via a mode measurement system utilizing a scanning laser vibrometer. This step is especially significant when the examined PEH has unknown parameters. Importantly, the measured mode can also be used for calculating the aerodynamic drag. The system is characterized in the following section.

### 2.11. Mode Measurement System

**Figure 2:**
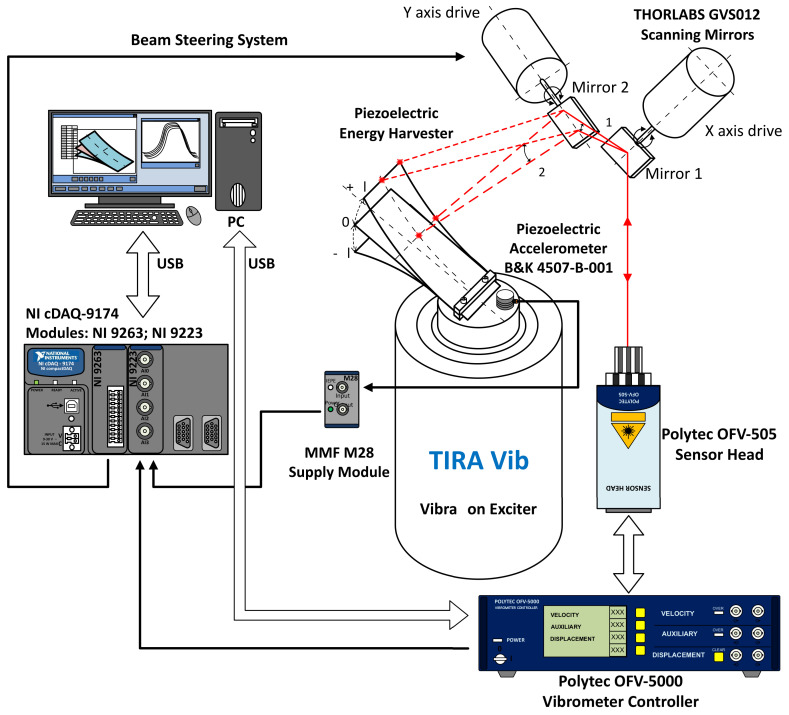
The scanning vibrometer setup to used verify that the first resonance mode has been reached.

The applied scanning laser vibrometer system (Figure 2) consists of a Polytec OFV-5000 vibrometer controller and Polytec OFV-505 sensor head, two-axis ThorLabs GVS012 scanning mirrors, a National Instruments Compact DAQ-9174 chassis with an NI 9223 analog input card (to ensure the acquisition of the measured and the reference vibrations), and an NI 9263 voltage output card (to adjust the mirrors’ positions).

The system is controlled by a custom-built, PC-based LabVIEW application, which defines and corrects the movement of the mirrors and the laser focus to measure at selected points on the PEH. In addition to these operations, the program acquires the vibrometer velocity data for every set point. The reference vibration was measured with a B&K 4507-B-001 accelerometer to enable the phase compensation of the discrete vibration measurements at the points selected on the PEH’s surface. This step ensures the determination of the vibration mode and allows the measurement of the acoustic losses. As the laser vibrometer acquires the velocity of every measured point, it was integrated into the spectral domain to yield the displacement amplitude. The obtained data can be used to confirm the resonance mode and to calculate the acoustic power produced by the PEH.

### 2.12. Efficiency Measurement System

**Figure 3:**
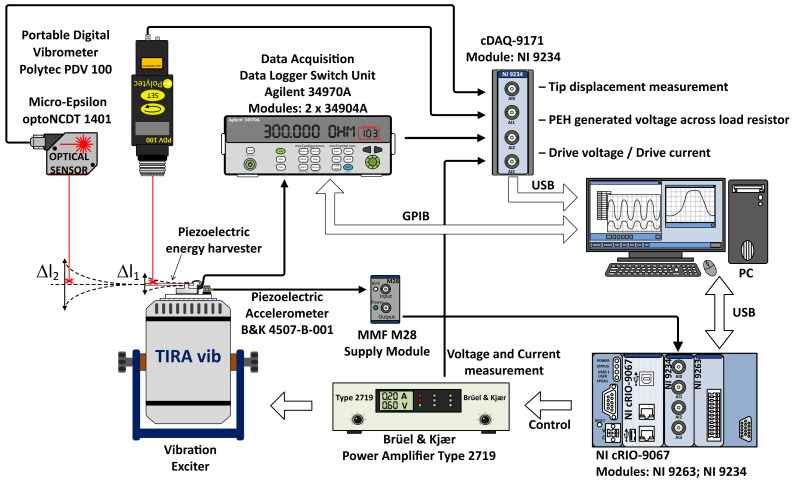
The system used to measure the power and efficiency.

The system comprises two parts (Figure 3), one measuring the harvesters’ parameters and the other controlling the vibrations. By definition, the measuring component sets the resistance via an Agilent 34970A data acquisition/switch unit and communicates the vibration parameters, frequency, and amplitude to its counterpart. Here, the vibrations are monitored and adjusted by a PI controller, and after they have settled down (error < 0.1% for 10 s, the system will measure the harvester’s output voltage on the pre-set load, the displacement of the harvester’s tip, and the shaker’s current and voltage. The acceleration data are then transferred from the vibration control unit. The smaller displacement amplitudes are measured by a Polytec PDV 100 vibrometer, while in the higher ones a Micro-Epsilon optoNCDT 1401-20 optical sensor is employed. The measurement is performed with a 24-bit, ±5 V, NI 9234 analog input card or, if the measured harvester’s output voltage reaches above 5 V, a 24-bit, ±30 V NI 9232 card. To minimize the noise (<40 μV and 127 μV, respectively) in the experiment, we selected sampling frequencies exhibiting the maximum decimation rate of 256. Moreover, the noise and possible scalloping loss were further reduced by extracting the effective value from the signal spectrum via an FFT with a flat top window [24].

The vibration control is ensured by a Compact RIO 9067 device equipped with NI 9234 analog input and NI 9263 analog output cards. The generated signal is transmitted to a B&K 2719 power amplifier, which drives a TIRA 52110 vibration shaker. The vibrations are measured by the B&K 4507-B-001 accelerometer, and the signal returns to the Compact RIO via an MFF M28 supply module. The accuracies of the accelerometer and the MMF supply module correspond to 0.4% (in the measured range of 20–300 Hz) and 0.5%, respectively; both of the units are calibrated. To reduce the noise, we employed the method characterized above.

The resistive load is configured by a set of different parallel combinations of resistors on an Agilent 34904A matrix switch inserted into the Agilent 34970A measurement unit. Each of the two switches contains nine resistors, whose parallel combinations cover two decades of resistance (meaning that four decades in total are covered). Throughout the measurement process, the resistances are controlled by an Agilent 34401A multimeter, with the repeatability of the measurements amounting to less than 0.04%. When the parallel combination of the load and the measurement card input resistances produces a significant error, a voltage follower is employed. The entire system is located in an air-conditioned laboratory, where all the measurements take place.

### 2.13. Comparison with the Methodology Currently in Use

To facilitate comparison of the proposed efficiency measurement technique with the existing methodology, we calculated the efficiency values in the PEHs at the devices’ resonant frequencies and optimum loads by using the standard approach. Even though novel computing formulas are available, such as that described in [25], these share the same simplified principle, which interprets the harvester’s mechanical power as the input power. Thus, we decided to compare our new metrics with a well-established and widely used option (Equation (1)) [7] (simplified calculated). The procedure embodied in Equation (1) was experimentally verified by, for instance, the authors of reference [6], whose efficiency measurement concept is also used herein for comparison (simplified measured).

The data enabling the calculation of the aforementioned efficiencies were gathered during merely one measurement, meaning that the error that arises from diverse conditions cannot occur.

## 3. Results and Discussion

**Figure 4:**
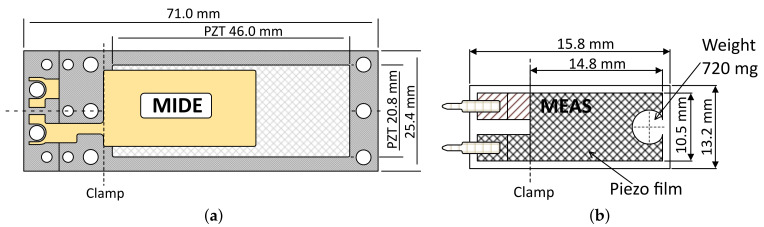
The tested PEHs: the MIDE PPA-1011 (**a**) and the TE LDTM-028K (**b**).

The measurement system was tested on two commercially available PEHs, one being a PZT PEH MIDE PPA-1011 (PZT PEH), which has a rigid structure with a FR4 base, and the other embodied in a PVDF TE LDTM-028K (PVDF PEH), which exhibits a flexible foil structure with an integrated 720 mg tip mass. The dimensions of these PEHs are shown in Figure 4; the capacitances equal 90.5 nF and 485 pF, respectively.

**Figure 5:**
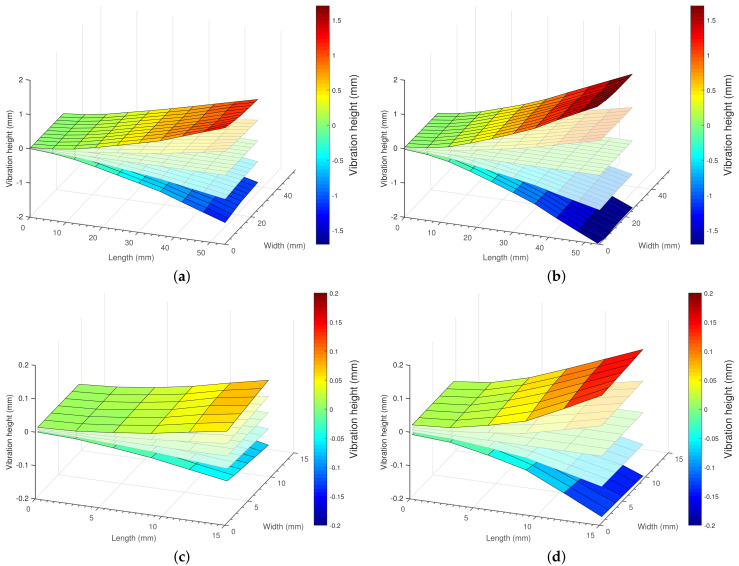
The confirmed oscillation: the first longitudinal mode in the MIDE PPA-1011 at 1 $g_{\mathrm{rms}}$ (**a**) and 2 $g_{\mathrm{rms}}$ (**b**); an in the TE LDTM-028K at 1 $g_{\mathrm{rms}}$ (**c**) and 2 $g_{\mathrm{rms}}$ (**d**).

**Figure 6:**
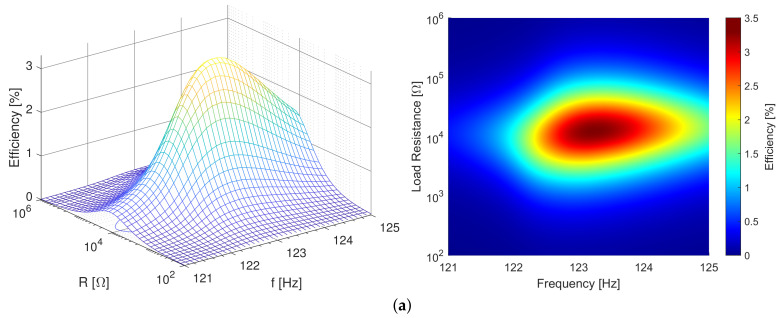

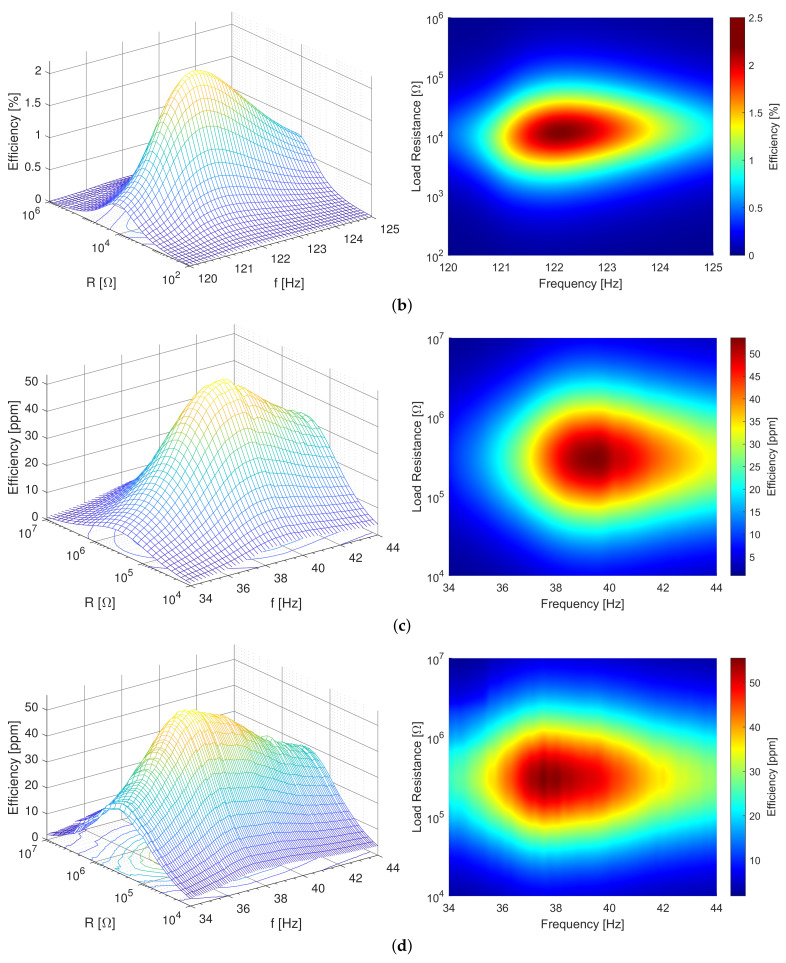
The proposed efficiency rates measured near the harvesters’ resonance frequencies, at 1 $g_{\mathrm{rms}}$ and 2 $g_{\mathrm{rms}}$: the zirconate titanate (PZT) PEH, (**a**,**b**, respectively); the polyvinylidene fluoride (PVDF) PEH, (**c**,**d**, respectively). The 3D plots and the top views are shown on the left-hand and the right-hand sides, respectively. Note the different efficiency scales for the two PEHs.

Initially, each resonance frequency was found via the sine sweep, and then the vibration mode (Figure 5) of the examined harvester was checked. The measured amplitudes enabled us to calculate the acoustic power corresponding to the aerodynamic drag. At the final stage, we carried out the same measurement cycles at different vibration amplitudes. The 3D proposed efficiency plots (side and top views) relating to selected vibration amplitudes are shown in Figure 6.

By common definition, the power generated by the PEHs increases with the vibration amplitude. In the PZT PEH, the power varied between 165 μW at 0.25 $g_{\mathrm{rms}}$ and 3.78 mW at 2 $g_{\mathrm{rms}}$. The PVDF PEH, however, is smaller and exhibits a higher optimal load, and thus the power values dropped to between 11.6 nW at 0.25 $g_{\mathrm{rms}}$ and 793 nW at 2 $g_{\mathrm{rms}}$.

### 3.1. Uncertainties of the Measurement System

The type B uncertainty was calculated analytically from the devices’ specifications. In this uncertainty, the cause consisted in the errors of the NI measurement cards and the accelerometer chain. The error distribution in the individual devices was considered normal (Gaussian), allowing us to calculate the uncertainty. The type B uncertainty varied from 0.003 to 0.4${\%}_{A}$ for the interval of 1σ. The unit of efficiency is usually %, and the same unit normally expresses relative error. In this paper, for clarity, the symbol ${\%}_{A}$ thus denotes the measured efficiency or its absolute error, whereas ${\%}_{R}$ stands for the relative error.

The type A uncertainty was determined as the worst case scenario from all measurements at different frequencies and vibration amplitudes, with each of these uncertainties established from 300 repetitive measuring cycles. The result to express the uncertainty was computed from the post-processed values. In general terms, this method involves the random measurement errors and their filtering by means of an FFT with a flat top window. The relative type A uncertainty amounted to 0.05, 0.03, and 0.002${\%}_{R}$ in the current, acceleration, and phase, respectively. The absolute combined uncertainty relating to the proposed efficiency measurement ranged between 0.007${\%}_{A}$ and 0.5 ${\%}_{A}$ for the interval of 1σ.

### 3.2. Uncertainty in Simplified Efficiencies

In the simplified measured and proposed efficiencies, the uncertainties were calculated similarly, from the measurement system’s uncertainties. The absolute combined uncertainty relating to the simplified measured efficiency varied between 0.015${\%}_{A}$ and 0.27${\%}_{A}$ for the interval of 1σ.

The uncertainty of the simplified calculated efficiency, however, arises from the uncertainty of the parameters $k_{\mathrm{sys}}$ and *Q*, which are calculated from relevant frequencies. For instance, in the PZT PEH, vibrating at 1 $g_{\mathrm{rms}}$, the open natural frequency attained $f_{o}=123.33$ Hz and the short one equaled $f_{s}=123.00$ Hz, both at the relative uncertainty 0.12${\%}_{R}$. The two frequencies being close to each other, the relative uncertainty of $k_{\mathrm{sys}}$ amounted to 63.3${\%}_{R}$. This rate, above all, then caused the combined absolute uncertainty of the simplified calculated efficiency to be much higher than those in the measured efficiencies, namely, it ranged between 3.05 and 5.56${\%}_{A}$ for the interval of 1σ.

### 3.3. Efficiency of the PZT Ceramic Based Harvester

**Figure 7:**
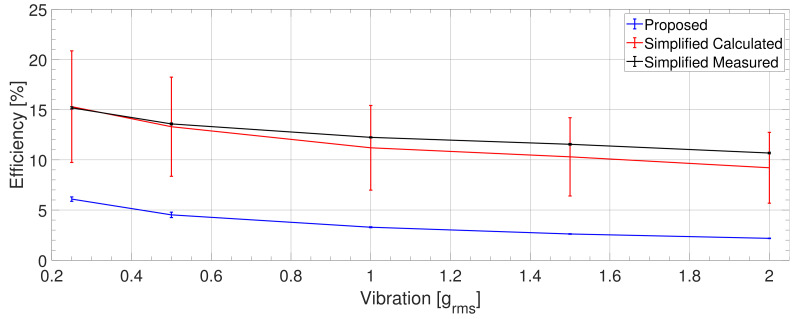
The PZT PEH: Comparing the efficiency values established via our method (proposed) with those calculated from the PEH’s parameters for the optimum condition (simplified calculated) and measured from the PEH’s mechanical power (simplified measured). The error bars represent the 1σ interval.

The diagram characterizing the PZT PEH (Figure 7) indicates that the simplified measured and simplified calculated efficiencies yielded similar results; the measured one, however, has a markedly lower uncertainty. These efficiencies are three to five times greater than the proposed efficiency because they did not take into account the mechanical losses.

Moreover, all types of efficiency exhibited a decreasing trend when the vibration amplitude rose. The proposed class nevertheless declined slightly faster due to the growth of the mechanical losses, which occurs with increasing vibration amplitude.

**Figure 8:**
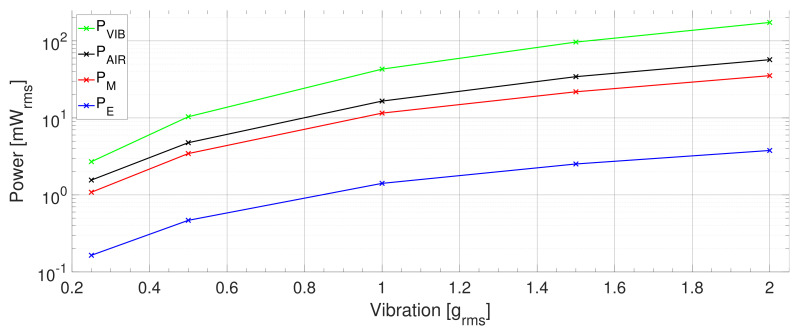
The PZT PEH: Comparing the power delivered by the shaker to the PEH, with the power dissipated in the air, the PEH mechanical vibration power, and the electrical power output.

In the given context, Figure 8 shows the individual powers of the PEH. It is worth noting that the aerodynamic loss was higher than the PEH’s mechanical power, the difference being approximately 50%. Furthermore, the amount of the vibration input power ($P_{\mathrm{VIB}}$) transferred to the mechanical power of the PEH ($P_{M}$) decreased with increasing vibration amplitude from approx. 40% to 20%. This indicates that the mechanical losses increased with rising vibration amplitude.

### 3.4. Efficiency of the PVDF Foil Based Harvester

**Figure 9:**
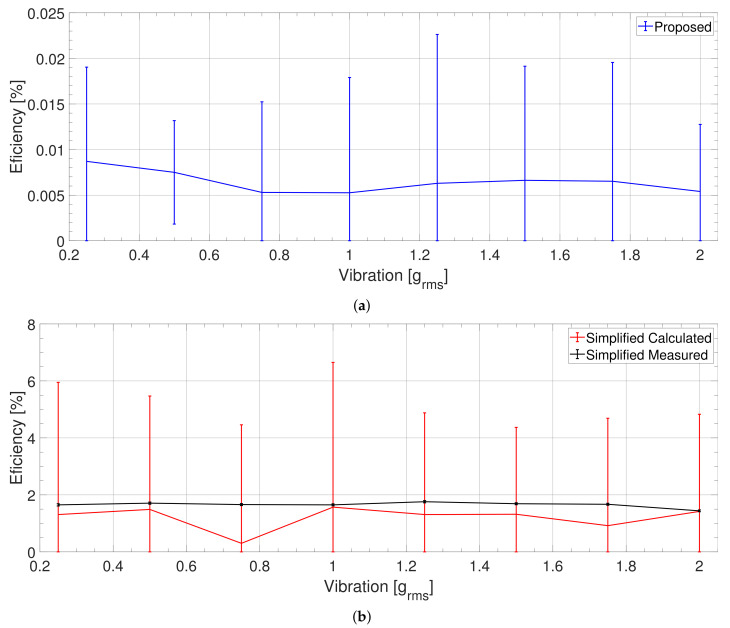
The PVDF PEH: Comparing the efficiency values established via our method (proposed) (**a**) with those calculated from the PEH’s parameters for the optimum condition (simplified calculation) and measured from the PEH mechanical power (simplified measurement) (**b**). The error bars represent the 1σ interval.

**Figure 10:**
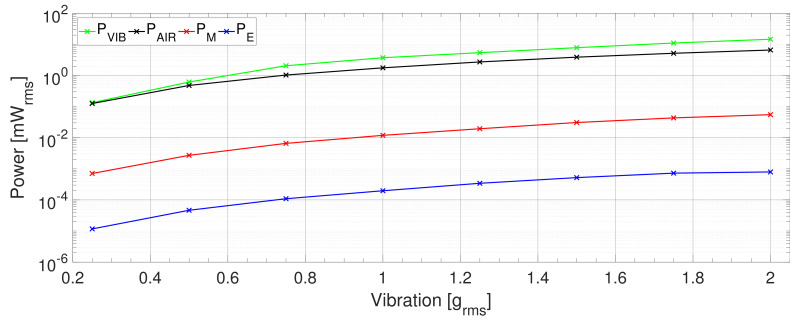
The PVDF PEH: Comparing the power delivered by the shaker to the PEH with the power dissipated in the air, the PEH mechanical vibration power, and the electrical power output.

In the PVDF PEH, the simplified calculated and simplified measured efficiencies (Figure 9) amounted to approximately 1.5${\%}_{A}$; the proposed efficiency was significantly smaller, varying between 0.005 and 0.015${\%}_{A}$. Such a condition is caused mainly by the harvester’s low stiffness, an effect stemming from the flexible structure. Thus, even though the displacement of the PEH’s tip is notable, the vibration power remained very small, being less than 1${\%}_{R}$ of the input vibration power (Figure 10). Consequently, due the major displacement, the aerodynamic losses produced more than a half of the overall ones. Moreover, the rather high optimum load resistance (330 kΩ) means that the power output was small, ranging between 11.6 nW at 0.25 $g_{\mathrm{rms}}$ and 793 nW at 2 $g_{\mathrm{rms}}$.

### 3.5. Comparing the Efficiencies

The simplified efficiencies (measured and calculated) exhibited the same results, similarly to the findings presented by Yang et al. [6].

The proposed efficiency showed lower values because the mechanical losses not only need to be taken into account but are also, as is obvious from the measured data, higher than the actual mechanical power of the PEH ($P_{M}$). In general terms, it is then possible to claim that the mechanical losses embody a significant factor affecting the performance of PEHs. Ignoring the losses during in the input power calculations involved in the computation of the simplified efficiency computations of the PEH can cause a significant error when different types of PEHs are compared for various purposes. Our novel method enables direct efficiency measurement and does not omit the mechanical losses of the PEH.

Even in situations where the mechanical losses can be neglected, the calculation of the efficiency from the parameters (1) is affected by a large uncertainty. This arises mainly from the uncertainty of the system coupling coefficient ($k_{\mathrm{sys}}$). If the same efficiency is measured via the PEH’s mechanical power ($P_{M}$), the uncertainty is significantly lower.

Moreover, excluding the harvester’s parameters allows us to use the proposed method for comparing different principles of power generation from ambient vibrations. Our system is capable of measuring a wide range of efficiencies, from tens of percent to units of parts per million, and the shape of the data (Figure 6) appears to be credible even at low efficiency rates. The uncertainty of the measured efficiency is also provided.

### 3.6. Power Flow

Once the PEH has been characterized, we can determine the individual power components according to the classification in Figure 1. The individual power components in the absolute value and their contribution to the total power consumed by the PEH are shown in Table 1. These items of information allow us to localize the most significant losses in the power conversion.

The most significant types of loss were the acoustic and thermal ones, which together dissipated more than two thirds of the total power input. Regrettably, we were able to find only a few papers that focused on reducing such losses [26,27,28]. By contrast, many research teams are developing new piezomaterials with the aim to reduce the internal losses; these processes, however, dissipate only a small portion of the power input. Finally, the electrical losses are negligible when compared to the other categories. The intensive research and development of power conditioning circuits will nevertheless allow the electrical losses to be reduced even further in the future.

In conclusion, let us emphasize again that multiple efforts are being made to minimize the internal and electrical losses (which embody only a small portion of the total losses), whereas the type of loss that exhibits the maximum contribution to the total sum remains mostly ignored.

## 4. Conclusions

By reviewing the papers that discuss techniques for calculating the efficiency of PEHs, we concluded that researchers regularly compute the input power as the power stored in the harvester’s movement. Such a procedure, however, causes the mechanical losses induced by the actual motion to be ignored. We designed a method to measure the efficiency of energy harvesters in such a manner that the mechanical losses generated by internal friction and aerodynamic drag are taken into account. Unlike the traditional approach, our technique relies on computing the input power out of the vibration power extracted from the shaker, with the losses preserved. In the experiments, a mode measurement system was employed to estimate the losses linked to the aerodynamic drag.

We developed a system capable of characterizing the power and measuring directly the overall efficiency of a harvester; the combined absolute uncertainty amounted to less than 0.5${\%}_{A}$. The measurement system was tested on commercially available PZT and PVDF PEHs: a MIDE PPA-1011 and a TE LDTM-028K. In the former, the efficiency at the resonant frequency and optimal load varied from 6.1${\%}_{A}$ at 0.25 $g_{\mathrm{rms}}$ to 2.2${\%}_{A}$ at 2 $g_{\mathrm{rms}}$. In the latter, the same quantity reached approximately 0.007${\%}_{A}$ in all of the measured vibration amplitudes (0.25–2 $g_{\mathrm{rms}}$).

The efficiency data relating to the optimum load and frequency were compared with the calculations based on the PEHs’ coupling coefficients and quality factors. In the PZT harvester, the proposed measured efficiency rate was roughly three to five times lower than the efficiencies determined via the existing methodology. The PVDF device, however, exhibited rates markedly lower than those established in the PZT one. Generally, it is then possible to claim that the mechanical losses embody a significant factor affecting the performance of PEHs.

We localized the main sources of loss, identifying these with the thermal and acoustic losses caused by the movement of the PEH. The internal losses, arising from the piezoelectric efficiency, consist of only a small portion of the total dissipated power, and the electrical ones, generated by the internal resistance, are negligible. Regrettably, while efforts are being made to minimize those sources of loss that exert a minimum impact on the total losses, only a few research groups focus on the major sources.

As our metrics are independent of the PEHs’ parameters, they can be applied in comparing the efficiencies of diverse vibration harvesters. The proposed method may accelerate the development of new vibration harvesters in general because the partial procedures comprised within the technique allow easy and accurate measurement of the devices’ performances. By extension, as suggested above, an interesting benefit lies in the possibility of comparing the outcomes delivered by different research teams.

In terms of future research, we intend to improve the measurement system to better measure and model the individual types of loss. Such an aim should then produce an increase in the efficiency of piezo energy harvesters.

## Figures and Tables

**Table 1:** Comparing the individual power components in the tested PEHs at 1 $g_{\mathrm{rms}}$.

Power Type		MIDE PPA-1011		TE LDTM-028K	
		[mW]	[%]	[μW]	[%]
Vibration power	$P_{\mathrm{VIB}}$	43.04	100	3745	100
→ Acoustic losses	$P_{\mathrm{AIR}}$	16.56	38.5	1770	47.3
→ Thermal losses	$P_{\mathrm{TH}}$	14.93	34.7	1963	52.4
→ PEH mechanical power	$P_{M}$	11.55	26.8	11.94	0.318
→ Internal losses	$P_{\mathrm{IL}}$	8.72	20.2	11.55	0.308
→ Electrical losses	$P_{\mathrm{EL}}$	1.41	3.3	0.197	0.005
→ Electrical power output	$P_{E}$	1.41	3.3	0.197	0.005

## Data Availability

The data used for the manuscript are available for researchers on request.

## Abbreviations

The following abbreviations are used in this manuscript:

PEH	Piezoelectric Energy Harvester
PZT	Lead Zirconate Titanate
PVDF	Polyvinylidene Fluoride
$k_{\mathrm{sys}}$	System Coupling Coefficient
Q	Quality Factor
$f_{o}$	Open Circuit Natural Frequency
$f_{s}$	Short Circuit Natural Frequency
$P_{\mathrm{SH}}$	Shaker Vibration Power
$P_{{\mathrm{SH}}_{\mathrm{PEH}}}$	Shaker Vibration Power with Mounted PEH
$P_{{\mathrm{SH}}_{\mathrm{NO}}}$	Shaker Vibration Power without PEH
$P_{\mathrm{VIB}}$	Vibration Power
$P_{\mathrm{AIR}}$	Acoustic Losses
$P_{\mathrm{TH}}$	Thermal Losses
$P_{M}$	PEH Mechanical Power
$P_{\mathrm{IL}}$	Internal Losses
$P_{\mathrm{EL}}$	Electrical Losses
$P_{E}$	Electrical Power Output
*F*	Vibration Force
*v*	Vibration Velocity
$k_{BI}$	Shaker Bl Coefficient
*a*	Vibration Amplitude
*f*	Frequency of Vibration
${\%}_{A}$	Percent as Absolute Quantity (used for the efficiency value and its absolute error)
${\%}_{R}$	Percent as Relative Quantity (used for the relative error)
